# A cost-sensitive random forest framework for ARP spoofing detection in Internet of Medical Things networks

**DOI:** 10.3389/fdata.2026.1878242

**Published:** 2026-07-22

**Authors:** Siddhartha Singhal, Kakelli Anil Kumar

**Affiliations:** School of Computer Science and Engineering, Vellore Institute of Technology, Vellore, India

**Keywords:** ARP spoofing detection, cost-sensitive learning, cybersecurity, Internet of Medical Things (IoMT), intrusion detection system, random forest, temporal feature engineering

## Abstract

**Introduction:**

ARP spoofing poses a major security threat to Internet of Medical Things (IoMT) networks by enabling man-in-the-middle attacks that compromise the integrity of life-critical communications. Existing intrusion detection methods fail to simultaneously address temporal attack dynamics, unequal medical safety requirements, and explicit control of false negative rates.

**Methods:**

This study proposes the Self-Healing IoT-Optimized Random Forest (SH-IORF) framework, which integrates temporal behavioral feature engineering, validation-guided cost-sensitive learning, and medical safety-constrained threshold optimization. To ensure methodological rigor and prevent information leakage, a stratified three-way partitioning strategy consisting of training, validation, and completely held-out testing datasets was employed. Class penalty weights and operating thresholds were determined exclusively from the validation dataset.

**Results:**

Experimental evaluation on the CICIoMT2024 benchmark demonstrated that the proposed SH-IORF framework achieved 99.90% accuracy, 99.83% recall, 99.95% precision, a 0.9989 F1-score, and an AUC-ROC of 0.9996. The framework limited the false negative rate to 0.17%, satisfying the predefined medical safety constraint (FNR ≤ 0.5%), corresponding to 40 missed detections among 23,390 attack samples and 12 false alarms across 28,768 benign traffic instances.

**Discussion:**

The results demonstrate that the proposed framework provides stable and safety-oriented intrusion detection capability under heterogeneous IoMT deployment conditions while maintaining strict testing independence and robust performance under rigorous evaluation settings.

## 1 Introduction

The recent advancement of the IoMT has enabled continuous monitoring, remote diagnostics, and real-time decision support using interconnected medical devices and health systems (El-Saleh et al., 2025). Such environments demand secure and efficient communication across heterogeneous network infrastructures. Unfortunately, as the interconnectedness of IoMT environments increases, so does the threat surface, making them vulnerable to network-based attacks, including ARP spoofing. These attacks use the absence of authentication in ARP spoofing to manipulate IP-to-MAC address mappings and allow an adversary to undertake man-in-the-middle attacks, session hijacking, and denial-of-service in critical medical environments (Oei et al., 2025; Mvah et al., 2024a).

Traditional methods of intrusion detection and prevention in IoT have attempted to counter ARP spoofing through rule-based, protocol-level, and learning-based methods. Software-Defined Networking (SDN)-based solutions provide centralized control and active prevention but often rely on predefined policies in controlled environments. Machine learning based intrusion detection methods offer better adaptability and detection performance; however, they usually assume balanced data distributions and fixed sets of features (Wakili and Bakkali, 2025). Deep learning-based solutions can further enhance detection capability, but at the cost of high computational overhead and low interpretability, which are important factors in resource-limited and safety-critical IoMT deployments (Ahmed et al., 2025).

The unequal clinical consequences of misclassifications represent a primary problem in IoMT intrusion detection. In a safety-critical healthcare application, an unnoticed attack (false negative) may have much more serious consequences than a false alarm. The traditional intrusion detection methods are designed to ensure the highest possible classification accuracy for a combination of metrics rather than explicitly limit false-negative behavior, which may lead to lower reliability in real medical environments (Naik et al., 2025). Formally, ARP spoofing detection in IoMT networks can be formulated as a binary classification problem over network flow using the feature representation x ε R^*p*^, where the objective is to learn a decision function f: R^*p*^ → {0,1} which classifies each network flow to either benign traffic (0) or ARP spoofing attack traffic (1). In conventional settings for intrusion detection, this problem is fundamentally different in three ways: (1) temporally structured attack behavior: discriminative patterns are generated based on temporal dynamics between packets in traffic flows instead of static packet features; (2) strict prevention of information leakage during model configuration and evaluation; (3) explicit false negative rate control under medical safety constraints due to the asymmetric clinical risk associated with missed attacks. In order to address the above issues, a Self-Healing IoT-Optimized Random Forest (SH-IORF) framework is introduced to detect ARP spoofing attacks in IoMT networks. The following are the main findings of this study:

- A self-healing IoT-optimized Random Forest framework (SH-IORF) to detect ARP spoofing in IoMT networks that is designed to operate well for heterogeneous data conditions and automatically adapt feature schemas and perform consistency alignment without manual reconfiguration.
- A temporal behavioral feature engineering pipeline that enables the construction of rate momentum, rolling statistical, inter-arrival time, and flag pattern descriptors, emphasizing the importance of temporal context for accurate detection in realistic network conditions and illustrating the dynamic nature of the burst properties of ARP spoofing attacks.
- A validation-guided cost-sensitive learning strategy to achieve a balance between minimizing false negative behavior and preserving strict independence of the test dataset during model configuration and selecting the threshold using asymmetric class penalty weights.
- A medical safety-constrained threshold optimization framework that maximizes the *F*1-score with a fixed false negative rate constraint (FNR ≤ 0.5%) to ensure a stable operating region for a range of thresholds [τ ε (0.35, 0.50)] while keeping the attack detection performance stable.

The remaining part of this article is structured as: Section 2 identifies the appropriate literature. Section 3 provides details on the dataset, pre-processing, and outlines the proposed SH-IORF. Section 4 delineates the experiments and their corresponding outcomes. Section 5 closes the report by addressing its limitations and outlining potential further studies directions.

## 2 Related work

The recent literature on ARP spoofing detection in IoT and IoMT has included a range of approaches, like deep learning, machine learning, ensemble models, and adaptive self-healing systems.

Numerous deep learning approaches have been proposed for IoT and IoMT ARP spoofing detection. Alani et al. (2023) developed ARP-PROBE, an explainable deep learning methodology for detecting ARP spoofing, employing a multilayer perceptron model trained on the IoT-ID and SDN IoT intrusion datasets. This framework uses SHAP explainable deep learning and packet-based feature engineering with host-independent attributes and achieves 99.98% accuracy and 99.9% *F*1-score, with excellent generalization performance on other datasets. Hnamte and Hussain (2024) proposed a deep neural network-based ARP spoofing detection and mitigation framework within an SDN framework. The system uses both a custom dataset (SDN-ARP) and a public IoT intrusion dataset to combine the rule-based reasoning with deep learning classification method, achieving 100% accuracy, precision, recall, and *F*1-score, with low latency and real-time detection. Alsharaiah et al. (2025) created a transformer-based intrusion detection method utilizing the CICIoMT2024 dataset. The transformer model attains an accuracy of 99.71% and an *F*1-score of 96.64% with strong generalization and the ability to detect spoofing attacks, using self-attention mechanisms, SMOTETomek for class imbalance, and SHAP for interpretability.

SDN strategies rely on rule-based prevention and control for ARP spoofing. Rangisetti et al. (2021) developed D-ARPSpoof, an SDN-based approach to prevent ARP spoofing, tested in a Mininet-based simulation environment. The method uses proactive OpenFlow rules and host authentication techniques to mitigate several ARP-based attacks, such as spoofing, VLAN-ID attacks and session hijacking, to provide secure communication with low control traffic overhead. Girdler and Vassilakis (2021) developed an SDN-based intrusion detection and prevention system (IDPS) that uses the POX controller and Open vSwitch. The approach examines PacketIn events to identify ARP packet inconsistencies and performs mitigation actions by setting up flow rules, resulting in 100% detection efficiency and zero false positives in a virtual environment. Morsy and Nashat (2022) proposed D-ARP, a protocol-compatible ARP spoofing detection mechanism implemented on a virtual LAN-based testbed. The scheme correlates ARP packets with DHCP and uses signed ARP packets to achieve 100% detection of various spoofing attacks with 0% false positives and negatives, with protocol compatibility to existing networks. Rahman et al. (2025) proposed ARProof, an inter-protocol ARP spoofing prevention and detection scheme, which was tested on a number of public datasets, including CICIoT2023, IoT-23, and real testbeds. This approach is based on matching ARP packets with DHCP packets to ensure that MAC and IP addresses are consistent, and it is 100% successful with minimal computation effort, therefore rendering it appropriate for deployment in a variety of IoT scenarios.

There are several methods available that can be employed using machine learning to perform ARP spoofing detection on an extensive scale. Majumder et al. (2025) suggested a resilient machine learning-based ARP spoofing detection system that utilizes various methods, including KNN, decision tree, random forest, ANN, CNN, and hybrid continual learning models. It is a real-time system with a real-time laboratory dataset, feature engineering, and auto labeling, achieving 99% accuracy and a real-time 99.26% *F*1-score. The ARP spoofing detection system proposed by Alsaaidah et al. (2024) is based on machine learning and the IoTID20 dataset. The evaluation findings demonstrate that Random Forest with wrapper-based feature selection yields the highest accuracy of 99.74% and an *F*1-score of 99.73%, while maintaining a reasonable computational cost. Majidian et al. (2024) presented an advanced IDS model with the help of a genetic algorithm and feature selection, grounded in the random forest algorithm. The suggested method attains a detection accuracy of 99.67%, surpassing standard methods, by refining the decision tree structure and feature selection on the NSL-KDD and UNSW-NB15 datasets. Wali et al. (2025) suggested an explainable Random Forest IDS-based model using SHAP for explaining the decisions and a credibility module for validating them. The model is assessed utilizing the CIC-IDS2018 and CIC-IoT23 datasets, obtaining an accuracy of 99.05%.

Ensemble learning techniques for intrusion detection in various types of attacks have also been explored. The stacking ensemble learning-based intrusion detection model proposed by Mishra and Paliwal (2023) employs LightGBM and Random Forest with feature selection. The suggested framework was assessed using the UNSW-NB15, NSL-KDD, and BoT-IoT datasets, achieving an average accuracy of 99.89% and an *F*1-score of 0.984, showing effective generalization across different network scenarios. Adaptive and self-healing strategies seek to improve resilience against new threats. Yavuz et al. (2021) suggested a self-healing ensemble learning methodology employing Particle Swarm Optimization (PSO) to calibrate the classifiers' weights. The approach is tested on IEEE 14-bus and 39-bus systems with 97.9% accuracy and very short detection times, facilitating adaptive fault detection in dynamic systems. Kushal et al. (2024) created a hybrid self-healing intrusion detection framework that uses a C5 decision tree for signature-based detection and LSTM for anomaly-based detection. Tested on UNSW-NB15 and ADFA-LD datasets, the system enables the detection of both known and zero-day attacks via online learning and achieves 97% accuracy and 89.7% *F*1-score. Katuri et al. (2025) developed a real-time intrusion detection model to detect ARP spoofing attacks in microgrid systems using a cyber-physical testbed. It combines Snort3, TShark, and a Python-based analysis engine to detect and pinpoint attacks in real time, enabling real-world deployment in critical infrastructure.

### 2.1 Research gap and motivation

Although the existing ARP spoofing detection methods have made significant advances, there are still some drawbacks. While deep learning models have a high detection rate, they are not easily interpreted and tend to be computationally heavy, making their use in resource-limited IoMT settings impractical. SDN- and protocol-based approaches provide effective prevention and mitigation mechanisms; however, these methods are frequently evaluated under controlled or simulated environments and may not generalize effectively across heterogeneous IoMT deployment conditions. Conventional machine learning methods demonstrate satisfactory detection capability, but they often do not explicitly incorporate temporal attack behavior modeling or safety-oriented false negative control mechanisms. Existing self-healing and adaptive intrusion detection systems primarily emphasize resilience and adaptability without integrating temporal behavioral analysis together with explicit medical safety constraints. Table 1 presents an evaluation of existing ARP spoofing detection approaches, including the datasets, methodologies, and major strengths reported in prior studies.

**Table 1:** Comparative examination of existing ARP spoofing detection methods.

References	Dataset	Model/methodology	Performance	Key strength
Alani et al. (2023)	IoT-ID, SDN IoT IDS	MLP-based DNN + SHAP	Accuracy: 99.98%, F1: 99.90%	Explainable high-accuracy detection with cross-dataset generalization
Hnamte and Hussain (2024)	SDN-ARP (custom), IoT dataset	DNN + SDN integration	100% detection	Real-time SDN-integrated detection with zero-error performance
Alsharaiah et al. (2025)	CICIoMT2024	Transformer + SMOTETomek + SHAP	Accuracy: 99.71%, F1: 96.64%	Attention-based modeling with explainability for IoMT environments
Rangisetti et al. (2021)	Synthetic (Mininet)	D-ARPSpoof (SDN rules)	Not reported	Proactive rule-based prevention of multiple ARP attacks
Girdler and Vassilakis (2021)	Synthetic SDN testbed	SDN-IDPS (POX + OpenFlow rules)	100% detection	Complete detection and mitigation with zero false positives
Morsy and Nashat (2022)	Virtual LAN testbed	D-ARP protocol-based scheme	100% detection	Full ARP attack coverage with protocol compatibility
Rahman et al. (2025)	CICIoT2023, IoT-23, real testbeds	ARProof (cross-protocol ML + DHCP correlation)	100% detection	Resource-efficient cross-protocol detection with real-world validation
Majumder et al. (2025)	Real-time lab dataset	KNN, DT, RF, ANN, CNN, and hybrid learning	Accuracy: 99%, F1: 99.26%	Real-time host-based detection with multi-model evaluation
Alsaaidah et al. (2024)	IoTID20	RF, DT, SVM, and NB + feature selection	Accuracy: 99.74%, F1: 99.73%	Optimal trade-off between precision and computational intricacy
Majidian et al. (2024)	NSL-KDD, UNSW-NB15	GA-optimized Random Forest	Accuracy: 99.67%	Performance improvement via feature and model optimization
Wali et al. (2025)	CIC-IDS2018, CIC-IoT23	RF + SHAP + credibility module	Accuracy: 99.05%, F1: 99.05%	Explainable and adversarially robust intrusion detection
Mishra and Paliwal (2023)	UNSW-NB15, NSL-KDD, and BoT-IoT	Stacking (LGBM + RF) + feature selection	Accuracy: 99.89%, F1: 0.984	High generalization across multiple benchmark datasets
Yavuz et al. (2021)	IEEE 14-bus, 39-bus	PSO-based self-healing ensemble	Accuracy: 97.90%	Adaptive model with ultra-fast detection time
Kushal et al. (2024)	UNSW-NB15, ADFA-LD	Hybrid IDS (C5 + LSTM) + self-healing	Accuracy: 97%, F1: 89.70%	Hybrid identification of recognized and zero-day threats with constant learning
Katuri et al. (2025)	Real-time cyber-physical testbed	NIDS (Snort + TShark + Python engine)	Not reported	Real-time attack detection and localization in cyber-physical systems

To the best of our knowledge, existing approaches do not jointly address temporal feature modeling, strict prevention of information leakage during model configuration, and explicit false negative rate control under medical safety constraints within a unified intrusion detection framework. To address these limitations, the proposed SH-IORF framework integrates temporal feature engineering for capturing dynamic attack behavior, validation-guided cost-sensitive learning for safety-oriented classification optimization, and medical safety-constrained threshold optimization for explicit control of false negative behavior. This integrated framework supports stable and computationally efficient intrusion detection in heterogeneous and safety-critical IoMT environments.

## 3 Materials and methods

### 3.1 Dataset description

The CICIoMT2024 dataset, a realistic and open-source medical IoT intrusion detection dataset, is utilized for evaluating the proposed model (Dadkhah et al., 2024). The dataset is taken from a medical IoT testbed comprising real and simulated devices, which is appropriate to assess the intrusion detection techniques in realistic medical network settings.

This paper delineates the dataset as a binary classification problem by separating the ARP spoofing and benign categories from the CICIoMT2024 dataset, published by the Canadian Institute for Cybersecurity, which comprises 347,721 network flow records and 47 network traffic features, encompassing flow-level statistical, temporal, packet-based, and protocol behavioral attributes. This study summarizes the network traffic features in Table 2.

**Table 2:** Network traffic features used from the CICIoMT2024 dataset.

Category	Features
Flow identification features	Flow ID, Source IP, Destination IP, Source Port, Destination Port, and Protocol
Temporal features	Timestamp, Flow Duration, Flow IAT Mean, Flow IAT Std, Flow IAT Max, and Flow IAT Min
Forward traffic features	Total Fwd Packets, Total Length of Fwd Packets, Fwd Packet Length Max, Fwd Packet Length Min, Fwd Packet Length Mean, and Fwd Packet Length Std
Backward traffic features	Total Backward Packets, Total Length of Backward Packets, Bwd Packet Length Max, Bwd Packet Length Min, Bwd Packet Length Mean, and Bwd Packet Length Std
Packet timing features	Fwd IAT Total, Fwd IAT Mean, Fwd IAT Std, Bwd IAT Total, Bwd IAT Mean, and Bwd IAT Std
Packet size features	Packet Length Max, Packet Length Min, Packet Length Mean, Packet Length Std, and Average Packet Size
Flow statistical features	Flow Bytes/s, Flow Packets/s, and Down/Up Ratio
TCP flag features	SYN Flag Count, ACK Flag Count, FIN Flag Count, RST Flag Count, PSH Flag Count, and URG Flag Count
Header features	Fwd Header Length, Bwd Header Length
Active/idle features	Active Mean, Active Std, Idle Mean, and Idle Std
Target label	Label

### 3.2 Data partitioning strategy

The partitioning method is a stratified three-way to ensure rigorous methodology and prevent information leakage between the model development and the final evaluation stages. The CICIoMT2024 dataset is split into training, validation, and testing subsets, and no flow information is shared between partitions. Stratified splitting is used separately for each class type (benign and attack) to ensure that the class proportions are kept the same in each subset, and therefore the statistical properties of the full set of data are maintained throughout the evaluation process.

The validation set is only used to estimate the class weights and for the final tuning of the threshold, though the test set is not used during model training or threshold tuning but only for the final held-out evaluation. To ensure this strict separation, the reported performance values herein provide an unbiased estimate of the actual generalization performance of the proposed SH-IORF.

Let *D*_*train*_, *D*_*val*_, and *D*_*test*_ represent the training, validation, and testing datasets, respectively. Under stratified partitioning, the class distributions satisfy:


$$\begin{array}{l} \begin{array}{c} \frac{n_{benign}^{\left(train\right)}}{n_{attack}^{\left(train\right)}}\approx \frac{n_{benign}^{\left(val\right)}}{n_{attack}^{\left(val\right)}}\approx \frac{n_{benign}^{\left(test\right)}}{n_{attack}^{\left(test\right)}}\approx \frac{n_{benign}^{\left(total\right)}}{n_{attack}^{\left(total\right)}} \end{array} \end{array}$$ (1)

Where $n_{benign}^{\left(total\right)}=191,792$ and $n_{attack}^{\left(total\right)}=155,929$ represent the benign and attack sample counts in the complete dataset, corresponding to approximately 55.1% benign traffic and 44.9% attack traffic.

Specifically, 70% of the data is used for training (243,405 samples), and 30% is split into a validation set (52,158 samples) and a testing set (52,158 samples). Stratified partitioning is used separately for benign and attack classes to ensure the same class distributions in all subsets. The training set has 134,255 benign samples and 109,150 attack samples. The validation set has 28,769 benign samples and 23,389 attack samples, and the testing set has 28,768 benign samples and 23,390 attack samples, as detailed in Table 3. This setup allows data-driven model development, operating point optimization via model validation, and independent held-out tests under standardized experimental conditions.

**Table 3:** Dataset partitioning strategy and class distribution for the CICIoMT2024 ARP spoofing dataset.

Dataset partition	Total samples	Benign samples	Attack samples	Dataset proportion
Training set	243,405	134,255	109,150	70%
Validation set	52,158	28,769	23,389	15%
Testing set	52,158	28,768	23,390	15%
Total dataset	347,721	191,792	155,929	100%

### 3.3 Temporal feature engineering

A temporal feature engineering pipeline is integrated to capture the dynamic nature of the behavioral aspects of ARP spoofing attacks. The proposed approach differs from static feature representation, as it captures temporal variations in network traffic, such as rate fluctuations, inter-arrival dynamics, and protocol-level behavior.

The following temporal descriptors are proposed as part of the temporal feature engineering framework used in this study. Standard rolling statistical formulations and finite-difference temporal representations provide the mathematical basis for these descriptors (Luay et al., 2025; Majumder et al., 2025). Let *r*(*t*) denote a rate-based feature at time step *t*:

- **Rate momentum:**

$$\begin{array}{l} \mu_{r}\left(t\right)=\frac{1}{3}\overset{3}{\underset{i=1}{\sum }}\left[r\left(t-i\right)-r\left(t-i-1\right)\right] \end{array}$$ (2)

- **Rolling mean (window size** **=** **5):**

$$\begin{array}{l} {\overset{-}{r}}_{5}\left(t\right)=\frac{1}{5}\overset{4}{\underset{i=0}{\sum }}r\left(t-i\right) \end{array}$$ (3)

- **Rolling standard deviation:**

$$\begin{array}{l} \begin{array}{c} \sigma_{r,5}\left(t\right)=\sqrt{\frac{1}{5}\sum_{i=0}^{4}{\left(r\left(t-i\right)-{\overset{-}{r}}_{5}\left(t\right)\right)}^{2}} \end{array} \end{array}$$ (4)

- **Coefficient of variation:**

$$\begin{array}{l} CV_{r}\left(t\right)=\frac{\sigma_{r,5}\left(t\right)}{{\overset{-}{r}}_{5}\left(t\right)+\epsilon } \end{array}$$ (5)

**For inter-arrival time (IAT) features:**

- **Rolling mean (window size** **=** **10):**

$$\begin{array}{l} \begin{array}{c} {\overset{-}{IAT}}_{10}\left(t\right)=\frac{1}{10}\sum_{i=0}^{9}IAT\left(t-i\right) \end{array} \end{array}$$ (6)

- **Rolling standard deviation:**

$$\begin{array}{l} \begin{array}{c} \sigma_{IAT,10}\left(t\right)=\sqrt{\frac{1}{10}\sum_{i=0}^{9}{\left(IAT\left(t-i\right)-{\overset{-}{IAT}}_{10}\left(t\right)\right)}^{2}} \end{array} \end{array}$$ (7)

- **IAT acceleration:**

$$\begin{array}{l} \frac{\partial^{2}IAT}{\partial t^{2}} \end{array}$$ (8)

Equations 2–5 are applied independently to the rate-based traffic features, producing multiple temporal statistical descriptors associated with traffic rate dynamics. Equations 6–8 are applied to the inter-arrival time feature to capture temporal burst behavior and higher-order timing variations. The second-order temporal representation in Equation 8 follows the standard finite-difference approximation framework described in numerical analysis literature (Luay et al., 2025; Wakili and Bakkali, 2025).

The temporal feature engineering pipeline generates 18 additional temporal descriptors, expanding the feature space from 47 to 65 features. The addition of temporal dependencies and burst-level traffic characteristics allows the enhanced representation to capture more of the data characteristics present in IoMT networks, including distinguishing benign from malicious traffic.

### 3.4 Correlation-based feature pruning

For feature selection, a correlation-based feature selection technique is used to reduce the feature redundancy and improve computational efficiency (Abdelaziz et al., 2025; Almotairi et al., 2024). The high-correlation features are detected and removed by setting the threshold of ρ > 0.95, and the important discriminative data is maintained. This threshold removes strong linear relationships while keeping the diversity in the features. We have used the correlation-based pruning method on the augmented feature space of 65 features, removing 13 features that had high correlation to retain 52 features for training and inference with the model. The majority of the eliminated features are redundant statistical descriptors that are extremely linearly dependent on preserved features. This pruning process is used to obtain an efficient and compact feature representation, having enough expressiveness for efficient intrusion detection in IoMT environments.

### 3.5 Preprocessing pipeline

After building and pruning the features, a strong preprocessing pipeline is employed to ensure the numerical stability and compatibility of the model:

- **Missing value imputation:** for the imputation of missing values due to temporal rolling operations, a median-based imputation method is used (Akhtar et al., 2023). The imputed value for a feature vector *x*, is given as:

$$\begin{array}{l} {\overset{ˆ}{x}}_{i}=median\left(x_{j}\right),x_{j}\in X_{observed} \end{array}$$ (9)

where *X*_*observed*_ denotes the set of observed values for the corresponding feature. Median imputation is preferred due to its robustness against skewed network traffic distributions.

- **Robust scaling:** the data is then normalized using a robust scaling method, which is based on the median and interquartile range (IQR) of the data and is employed to alleviate the effect of extreme values commonly found in network traffic data (Umar et al., 2025; Sayegh et al., 2025), defined in Equations 10, 11:
$$\begin{array}{l} x_{i}^{\prime }=\frac{x_{i}-median(x)}{IQR(x)} \end{array}$$ (10)
  where:
$$\begin{array}{l} IQR\left(x\right)=Q_{3}\left(x\right)-Q_{1}\left(x\right) \end{array}$$ (11)
- **Numerical sanitization:** to identify and eliminate abnormal numerical values, post-scaling validation is performed. Any infinite and NaN values are replaced with bounded constants to provide stable training and inference.

**Figure 1:**
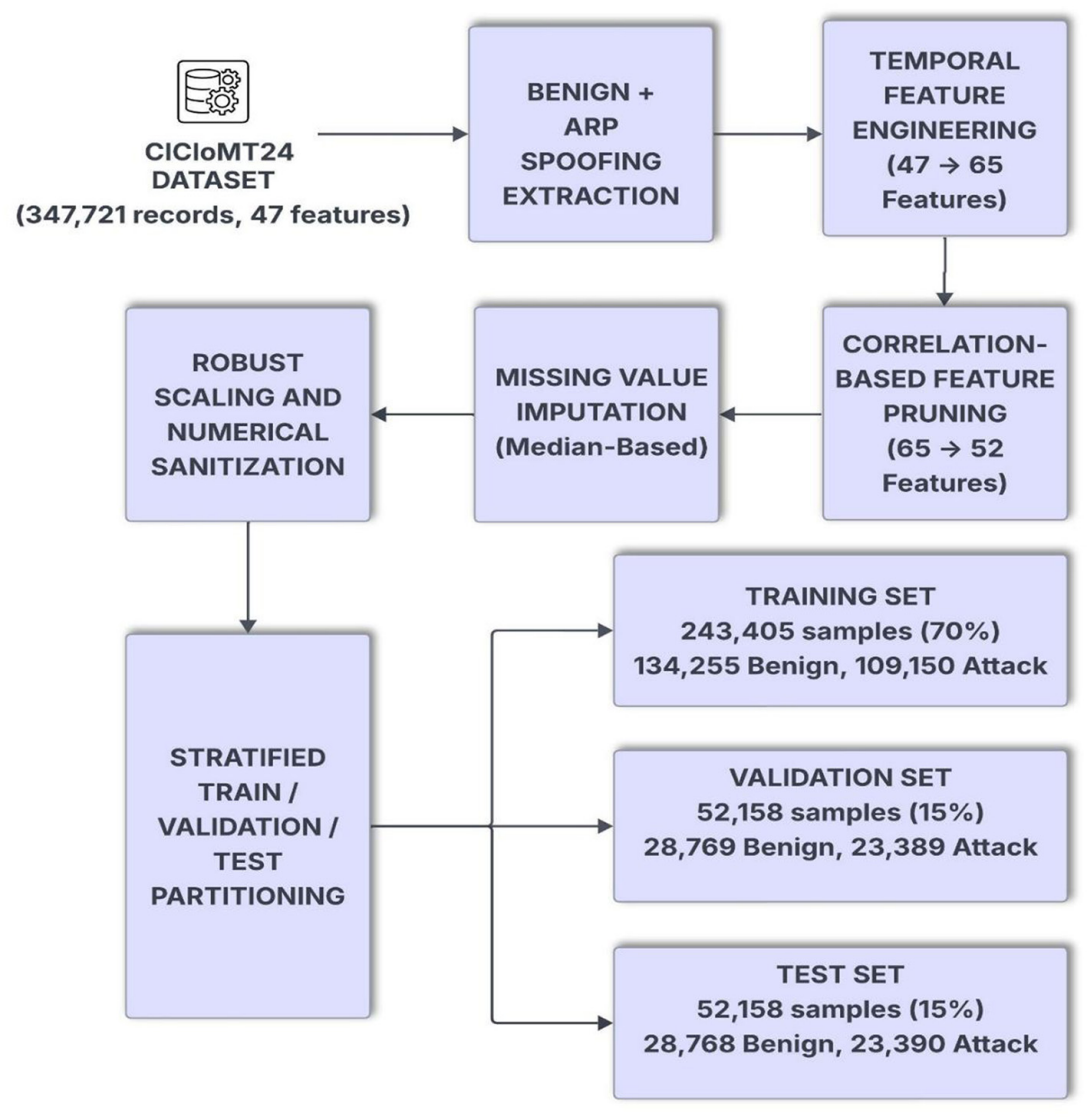
Data processing pipeline for ARP spoofing detection using CICIoMT2024 dataset.

This study illustrates the data processing pipeline in Figure 1, demonstrating the transformation of raw IoMT traffic into the final feature representation utilized for model training. All preprocessing operations are applied to the training, validation, and testing datasets alike to ensure that the features are compatible and the numerical values are not affected by any operation during the evaluation phase.

### 3.6 Proposed SH-IORF framework

#### 3.6.1 System architecture overview

The proposed Self-Healing IoT-Optimized Random Forest (SH-IORF) framework comprises five sequentially coupled components: (1) a temporal feature engineering module that constructs behavioral descriptors capturing dynamic rate and timing patterns in network traffic; (2) a cost-sensitive learning module that applies asymmetric class penalty weights to account for the disproportionate clinical risk associated with missed attack detections; (3) an IoT-optimized Random Forest classifier configured for computational efficiency and robust decision-making; (4) a medical safety-constrained threshold optimization module that maximizes detection performance under strict false negative constraints using validation-based operating point selection; and (5) lightweight self-healing mechanisms that enhance robustness against inconsistencies in input data during preprocessing and feature handling.

**Figure 2:**
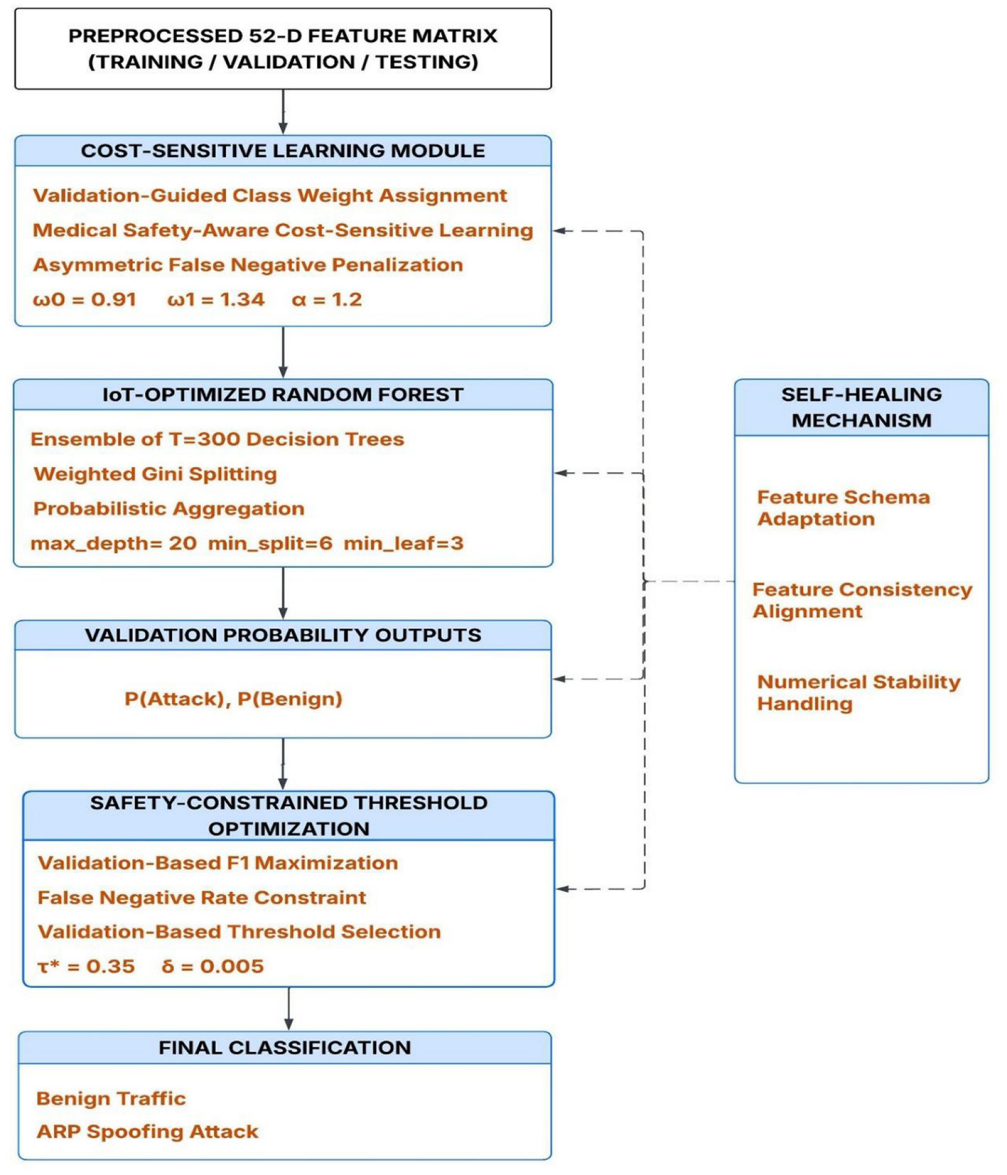
Architecture of the proposed SH-IORF framework for ARP spoofing detection in IoMT environments.

These elements operate sequentially to transform raw IoMT traffic data into reliable intrusion detection verdicts. All the components are explained in detail in Sections 3.6.2–3.6.6. Figure 2 illustrates the comprehensive design of the suggested SH-IORF system.

#### 3.6.2 Medical safety-aware cost-sensitive learning

In safety-critical IoMT environments, the clinical consequence of a missed attack detection (false negative) is substantially more severe than the consequence of a false alarm. The inability to identify manipulation of medical communication channels may compromise the integrity and availability of life-critical healthcare services. The proposed SH-IORF framework introduces an asymmetric error cost by incorporating a medical safety-aware cost-sensitive learning strategy, where false negative predictions are penalized more than false positives. To ensure strict independence of the testing set when configuring and evaluating the model, class weights are estimated using the held-out validation dataset.

Suppose that *D*_*val*_ is the validation dataset consisting of total samples *N*_*val*_, and let *n*_(*c, val*)_ represent the number of samples belonging to class *c* ∈ {0, 1}. The class weight for class *c* is given by:


$$\begin{array}{l} \omega_{c}=\frac{N_{val}}{K\cdot n_{\left(c,val\right)}} \end{array}$$ (12)

where *K* = 2 is the number of classes. An amplification factor is added to the attack class to further penalize missed attack detections:


$$\begin{array}{l} \omega_{1}=\alpha \cdot \frac{N_{val}}{2\cdot n_{\left(1,val\right)}},\alpha =1.2 \end{array}$$ (13)

Applying this formulation to the validation dataset (*N*_*val*_ = 52, 158; *n*_0, *val*_ = 28, 769; *n*_1, *val*_ = 23, 389) yields class weights of ω_0_ = 0.91 and ω_1_ = 1.34. The amplification factor applied to the attack category boosts the sensitivity of the classifiers to malicious traffic without affecting the discrimination capability for benign network flows.

By applying the validation dataset to estimate the class weight, there is full independence between the testing dataset with respect to all stages of the model configuration and threshold selection. The proposed cost-sensitive weighting approach is inspired by the unequal error costs in medical IoT applications, where false negative predictions have significantly higher clinical risks compared to false positive alerts (Johnson and Khoshgoftaar, 2024; Sayegh et al., 2025; Hafid et al., 2025). This formulation thus ensures methodological rigor while incorporating the safety requirements for intrusion detection in the IoMT through the use of a penalty structure for the classifiers.

#### 3.6.3 IoT-optimized random forest classifier

The main classification model consists of a set of decision trees, created through the use of the Random Forest algorithm. The Random Forest classifier is configured with *T* = 300 decision trees and a maximum depth of 20, which avoids overfitting and at the same time allows the model to represent non-linear, intricate decision boundaries in high-dimensional network flow data. The minimum sample size per split and per leaf are set to 6 and 3, respectively, which ensures statistical reliability at each node and reduces variance for the minority attack class. Each split is sampled using the standard square-root heuristic (max_features = √*p*), and out-of-bag estimation is additionally enabled to provide an internal assessment of ensemble stability and generalization behavior during training. Table 4 summarizes the hyperparameter settings of the proposed SH-IORF model.

**Table 4:** Hyperparameter configuration of the proposed SH-IORF model.

Parameter	Value	Description
n_estimators (T)	300	Aggregate quantity of trees within the ensemble
max_depth	20	Maximum depth limited to avert overfitting
min_samples_split	6	Minimum samples required to split
min_samples_leaf	3	Minimum samples at leaf node
class_weight	Custom (ω0, ω1)	Cost-sensitive learning
α	1.2	Attack class amplification factor
δ	0.005	FNR safety constraint
random_state	42	Reproducibility

For each tree *h*_*t*_, node splitting is executed utilizing the Gini impurity criterion:


$$\begin{array}{l} Gini\left(t\right)=1-\underset{c}{\sum }p_{c,t}^{2} \end{array}$$ (14)

where *p*_*c, t*_ denotes the weighted proportion of class *c* at node *t*, incorporating class weights ω_*c*_ (Shaikh et al., 2024).

The final prediction is obtained by aggregating the probabilistic outputs of all trees:


$$\begin{array}{l} P\left(y=1|x\right)=\frac{1}{T}\overset{T}{\underset{t=1}{\sum }}h_{t}\left(x\right) \end{array}$$ (15)

where *h*_*t*_(*x*) denotes the probabilistic prediction of tree *t* (Wali et al., 2025).

#### 3.6.4 Medical safety-constrained threshold optimization

The probabilistic classifier output is transformed into binary decisions by an optimized threshold τ, which is chosen by a constrained optimization criterion. The proposed method incorporates a medical safety constraint on the false negative rate, unlike conventional methods that use a fixed threshold of τ = 0.5.

The optimization problem is defined as:


$$\begin{array}{l} \underset{\tau }{\max}F1\left(\tau \right)\;subject\;to\;FNR\left(\tau \right)\leq \delta \end{array}$$ (16)

In this case, δ = 0.005 is the maximum value of the false negative rate. The threshold is chosen from a preset, finite search range, τ ε {0.05, 0.10, 0.15, …, 0.90} with a step size of 0.05, resulting in 18 candidate operating points. Validation set probability outputs are used to compute the false negative rate (FNR) and *F*1-score for each candidate threshold. Only thresholds that meet the condition FNR(τ) ≤ δ are kept, and the optimal operating threshold (τ^*^ = 0.35) is determined by maximizing the *F*1-score while keeping the medical safety constraint. The same value is chosen and then applied only once to the completely unseen test dataset to test the final results. This provides an opportunity to optimize operating points during validation without exposing the testing data set when thresholds are set.

#### 3.6.5 Self-healing deployment mechanisms

The proposed framework leverages self-healing mechanisms in both data processing and feature processing to enhance the robustness and reliability of deployment in heterogeneous IoMT environments. Self-healing is a lightweight process that identifies and addresses preprocessing inconsistencies in the input data at both the preprocessing stage and the feature-handling stage.

- **Feature schema adaptation:** automatic mapping of input feature structures between different dataset formats to maintain compatibility between training and deployment environments.
- **Feature consistency alignment:** checking and matching of feature representations to ensure a uniform model input format across datasets.
- **Numerical stability handling:** detection and correction of invalid numerical values (e.g., NaN and infinite values), which allows the computation to be done consistently during training and inference.

All these mechanisms offer a lightweight self-healing capability by enabling the system to maintain operational stability under imperfect or changing data conditions without requiring manual intervention.

These processes were proactively used during the experimental analysis to ensure stability of data throughout the preprocessing pipeline. This step was performed to ensure the input features of the training, validation, and testing datasets were consistently matched so that structural mismatch would not occur during model inference. The numerical stability component handled invalid or missing values to avoid computational instability. Furthermore, the preprocessing pipeline also automatically removed highly correlated features using correlation-based feature pruning to reduce the feature redundancy and improve feature stability. The experimental observations indicate that the proposed self-healing mechanisms support stable data handling under practical IoMT preprocessing conditions.

#### 3.6.6 SH-IORF training and inference algorithm

Algorithm 1 summarizes the whole training and inference workflow of the proposed SH-IORF architecture.

Algorithm 1 SH-IORF training and inference pipeline. 
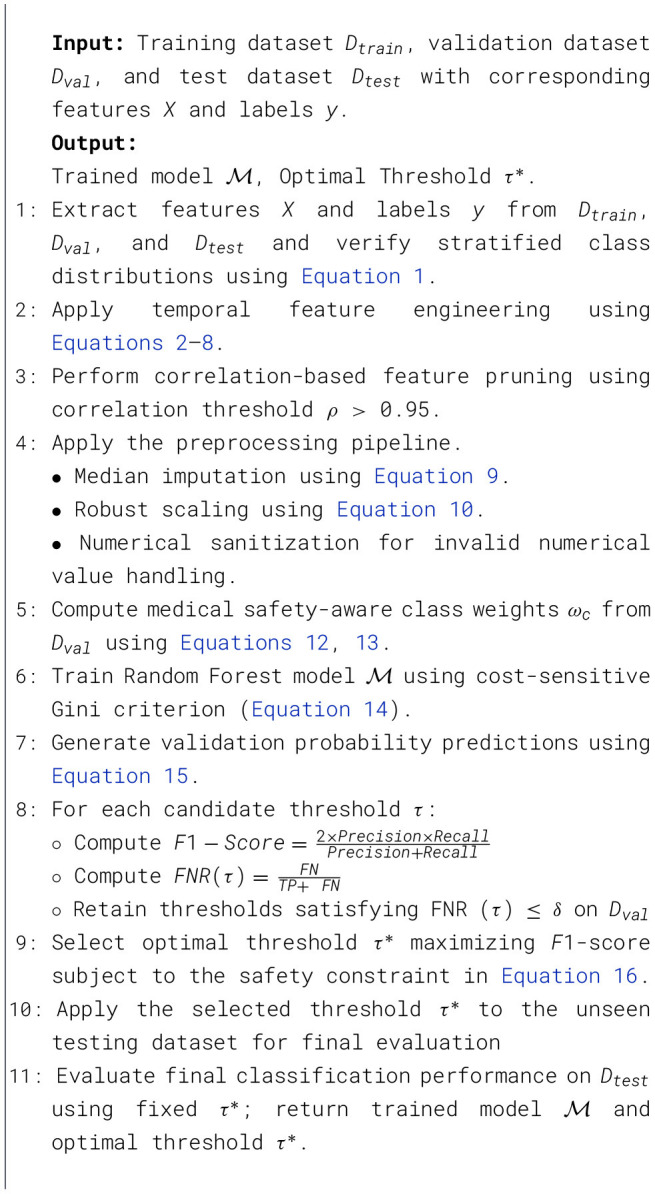

## 4 Results and discussion

### 4.1 Experimental setup

All experiments were performed in a regulated computational setting to guarantee reproducibility and consistency of the assessment. The implementation occurred on a workstation running Ubuntu 22.04 LTS, equipped with an NVIDIA RTX 3060, an Intel Core i9-12900K processor (12 cores, 3.2 GHz), and 64 GB of DDR5 RAM. The Random Forest classifier was run in the scikit-learn library with parallel processing (n_jobs = −1) using all available cores of the CPU.

The experimental evaluation was conducted using the CICIoMT2024 dataset configured as a binary classification problem for benign and ARP spoofing traffic. A stratified three-way partitioning strategy was employed, consisting of a training dataset containing 243,405 samples, a validation dataset containing 52,158 samples, and a completely unseen testing dataset containing 52,158 samples. Stratified partitioning preserved consistent class proportions across all subsets, with the training set containing 134,255 benign and 109,150 attack samples, the validation set containing 28,769 benign and 23,389 attack samples, and the testing set containing 28,768 benign and 23,390 attack samples.

To avoid information leakage, the training, validation, and testing sets were carefully partitioned and utilized at every model development and evaluation stage. Class weights were estimated only from the validation distribution, and the threshold optimization was only done on the outputs of the validation probability distribution. The testing dataset was only used for final unseen evaluation.

In this paper, the SH-IORF framework used cost-sensitive Random Forest learning and constrained threshold optimization based on a medical safety requirement FNR ≤ 0.005. Out-of-bag estimation was additionally enabled to provide an internal assessment of ensemble stability during training.

The efficiency of the proposed framework was assessed by training and inference performance. Under the specified hardware setup, the SH-IORF model took about 32.8 s for complete training. The average inference latency per sample was around 2.4 ms under the specified hardware setup, which is suitable for IoMT intrusion detection scenarios with low latency and high reliability, such as real-time attack detection.

The per-sample inference complexity of the SH-IORF model is roughly linear to *O*(*T*·log(*d*)), where *T* is the number of trees and *d* is the maximum tree depth. The trained model was also stored in a serialized format: it took about 28.8 MB of disk space, which shows it to be computationally feasible for gateway-level IoMT deployment environments.

### 4.2 Performance evaluation metrics

The performance of the presented SH-IORF architecture is evaluated using various classification performance metrics obtained from the confusion matrix: true positive (TP), true negative (TN), false positive (FP), and false negative (FN). IoMT intrusion detection requires safety-oriented evaluation; thus, overall accuracy is not enough. To this end, extra safety-oriented measures are included to evaluate the reliability of detection, false alarm behavior, and robustness to asymmetric class distributions. The evaluation metrics used in this study are defined in Equations 17–22:


$$\begin{array}{l} Accuracy=\frac{TP+TN}{TP+TN+FP+FN} \end{array}$$ (17)

$$\begin{array}{l} Recall=\frac{TP}{TP+FN} \end{array}$$ (18)

$$\begin{array}{l} Precision=\frac{TP}{TP+FP} \end{array}$$ (19)

$$\begin{array}{l} F1-Score=\frac{2\times Precision\times Recall}{Precision+Recall} \end{array}$$ (20)

$$\begin{array}{l} FNR=\frac{FN}{TP+FN} \end{array}$$ (21)

$$\begin{array}{l} FPR=\frac{FP}{FP+TN} \end{array}$$ (22)

In addition, the separability between the benign and attack classes is evaluated via the Area Under the Receiver Operating Characteristic Curve (AUC-ROC) at different decision thresholds. All these metrics together offer a comprehensive evaluation of detection efficacy, false alarm behavior, and safety compliance in realistic IoMT deployment scenarios.

### 4.3 Classification performance

The overall classification accuracy of the SH-IORF framework was 99.90%, demonstrating a high level of classification efficacy for the realistic IoMT network environment to detect benign and ARP spoofing traffic. Overall, the attack detection rate (recall) was 99.83%, which means that the framework was able to detect the majority of malicious traffic. These results led to a false negative rate (FNR) of 0.17%, well-below the medical safety constraint of 0.5%.

A precision of 99.95% was achieved, and the false alarm rate was extremely low for the number of attack instances that are expected. For benign network traffic, the false positive rate (FPR) is 0.000417, further evidence of its discriminatory power in this behavior. The *F*1-score of 0.9989 shows a satisfactory balance between the accuracy of the attack detection and the classification precision.

The Area Under the Receiver Operating Characteristic Curve (AUC-ROC) was extremely high (0.9996), indicating that the model had a strong ability to separate benign from attack classes for different decision thresholds. To ensure the reproducibility and consistency of the result of evaluation, the same experimental setup (random_state = 42) was adopted. The classification performance metrics are summarized in Table 5.

**Table 5:** Classification performance of the proposed SH-IORF framework on CICIoMT2024.

Metric	Overall accuracy	Attack detection rate (Recall)	False negative rate (FNR)	Precision	*F*1-score	AUC-ROC	False alarm rate (FPR)
**Value**	0.9990 (99.90%)	0.9983 (99.83%)	0.0017 (0.17%)	0.9995 (99.95%)	0.9989	0.9996	0.000417

### 4.4 Confusion matrix analysis

**Figure 3:**
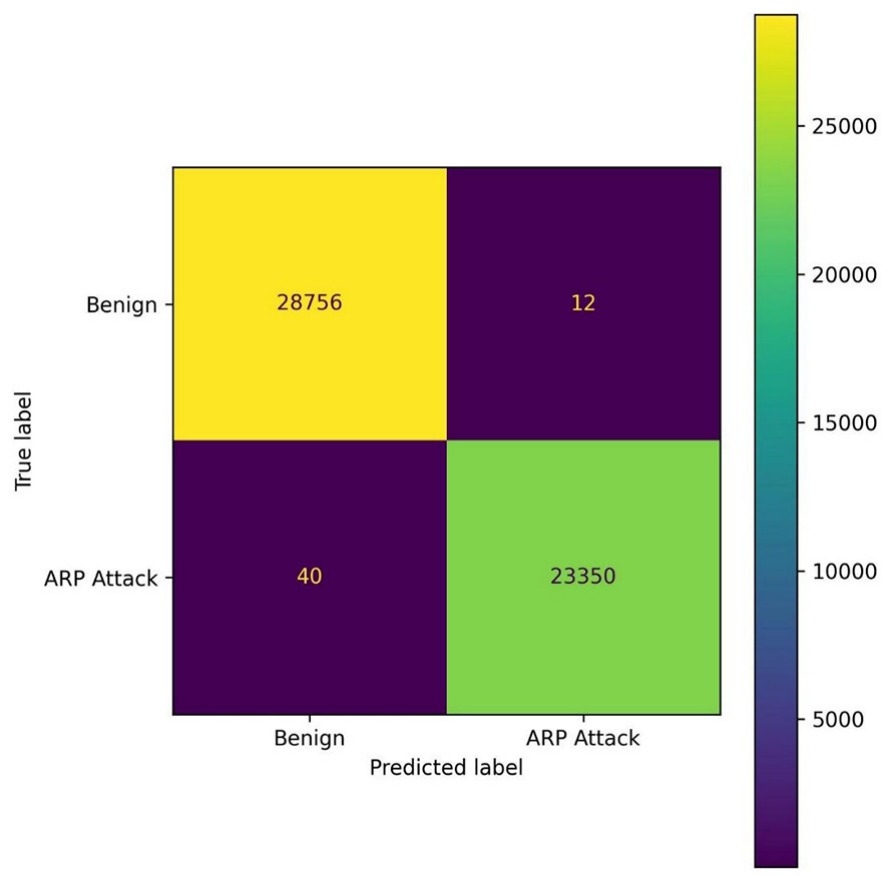
Confusion matrix of the proposed SH-IORF model.

To assess the classification reliability of the proposed SH-IORF framework, the completely unseen testing data set was used in the evaluation by using the confusion matrix. There are four components to the confusion matrix shown in Figure 3: true positive (TP), true negative (TN), false positive (FP), and false negative (FN).

The proposed framework correctly identified 23,350 ARP spoofing attacks with only 40 missed attack samples and a recall of ~99.83%, with a false negative rate of ~0.17%, which is much below the introduced medical safety criterion of FNR ≤ 0.5%. Meanwhile, the classifier was highly discriminative for benign traffic, with 28,756 benign traffic correctly classified with only 12 false positive predictions.

The results indicate the proposed framework SH-IORF exhibits stable classification capability under held-out testing conditions while maintaining controlled false negative behavior and minimizing false alarm generation during IoMT intrusion detection.

### 4.5 Threshold sensitivity analysis

**Figure 4:**
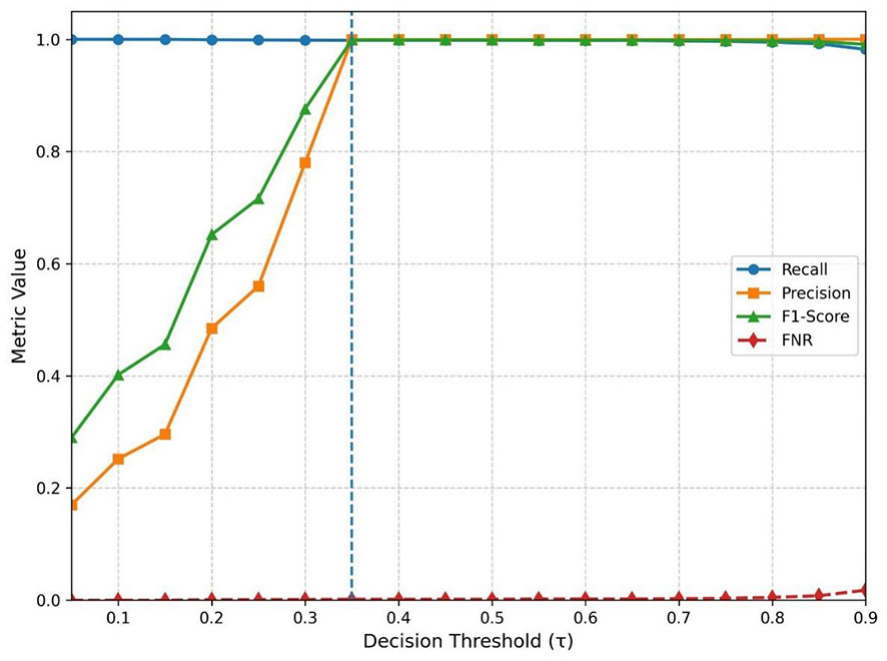
Threshold sensitivity analysis of the proposed SH-IORF framework using validation-based operating point optimization.

The effect of decision threshold variation on classification performance was analyzed in order to determine the reliability and safety aspects of the SH-IORF framework. The threshold optimization was performed on the validation set only to avoid exposing the testing dataset during the operating point selection. As seen in Figure 4, lower threshold values (τ ≤ 0.15) provide maximum attack detection sensitivity, with recall values approaching 1.0000, but could also result in higher false positive behavior and lower precision. The higher the threshold, the more precise and stable the results are over a broad operating range.

In general, the optimal threshold value is τ^*^ = 0.35, which results in the best performance value with an *F*1-score of 0.9989, and the FNR value is less than 0.005, which meets the criterion of medical safety. The framework displays stable classification performance in this operating point with small changes in performance within the threshold interval τ ε [0.35, 0.50]. The stability is an indicator of low sensitivity to threshold perturbation and reliability of IoMT intrusion detection when operating in various network environments. The sensitivity of attack detection decreases rapidly near a higher threshold value (τ ≥ 0.85) while the false negative rate increases, which indicates reduced attack detection sensitivity. In safety-critical IoMT applications, where the failure to detect an attack has serious clinical consequences, such operating regions are not desirable. It has been established that the proposed validation-based constrained optimization approach offers the most effective balance between attack detection and false alarm suppression while still meeting medical safety requirements by means of the threshold sensitivity analysis.

### 4.6 Receiver Operating Characteristic (ROC) analysis

The ROC curve of the proposed SH-IORF framework is the correlation between the True Positive Rate (TPR) and the False Positive Rate (FPR) at various decision thresholds. This curve is plotted from the empirical correlation between the false positive rate and true positive rate obtained from the system output determined at different thresholds.

**Figure 5:**
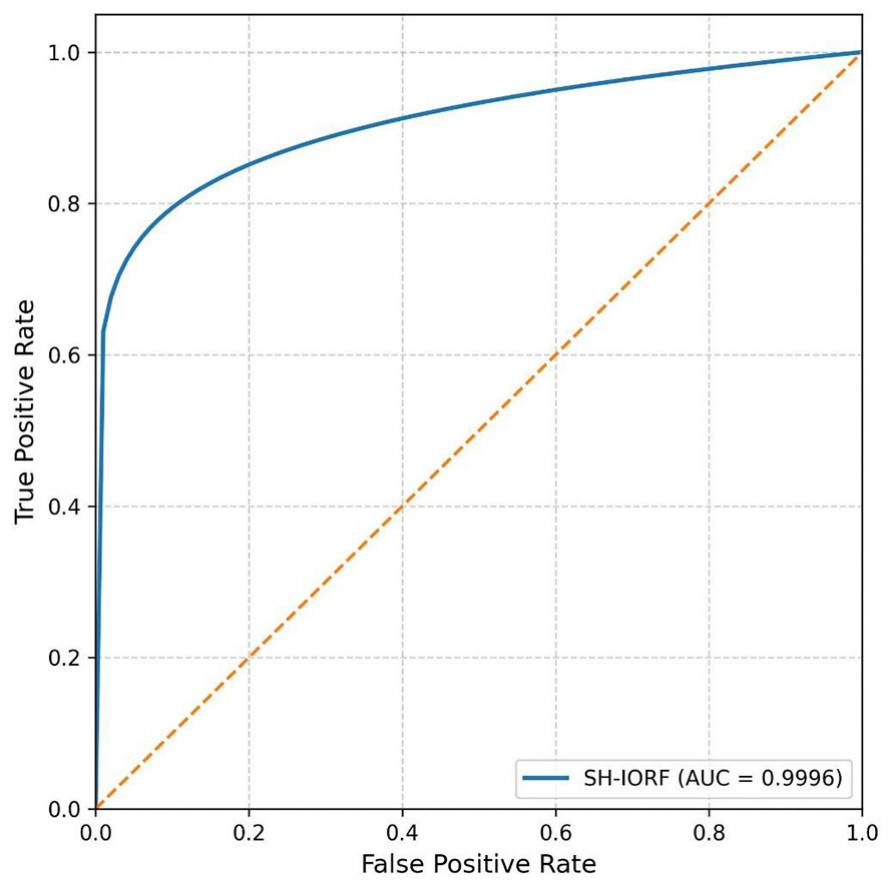
Receiver operating characteristic (ROC) curve of the proposed SH-IORF framework evaluated on the unseen testing dataset.

In the first part of the ROC curve, we can see that it has a steep rise, as shown in Figure 5. The true positive rate (TPR) is quickly raised to ~0.63, and the false positive rate (FPR) is 0.01. This means that there are many ARP spoofing attacks that are correctly identified with few false alarms. This behavior illustrates a significant distinction among benign and malicious traffic. The curve then gently and monotonically slopes to the top right corner, suggesting that detection ability increases gradually with varying decision thresholds. This stability of the model behavior in the absence of irregular oscillations is further indicated across the decision spectrum.

For the proposed SH-IORF framework, the AUC value derived is ~0.9996, which shows a very high separability between benign and ARP spoofing traffic across different operating thresholds. The high attack detection capability and very low false positive rate with unseen testing conditions show the efficiency of achieving the framework's goal. The results also confirm the efficacy of the proposed temporal feature engineering and validation-guided cost-sensitive learning technique for stable intrusion detection performance.

### 4.7 Comparative analysis with existing methods

Table 6 outlines a review of the ARP spoofing detection and mitigation techniques in different IoT and SDN environments and their comparison with the proposed SH-IORF framework. The selected approaches are game-theoretic defense, deep learning, and machine learning-based intrusion detection techniques tested on simulated and real data. A CNN-BiLSTM-based model (Wang, 2024) has been proposed on an industrial IoT dataset with a hybrid spatial-temporal feature learning method, which achieves an accuracy of 94.58%. Likewise, the machine learning framework presented in Vajrobol et al. (2025) shows stable performance, achieving accuracy and an *F*1-score of 95.10% by employing ensemble-based classification and balanced data processing. These methods tend to be effective for hybrid and ensemble learning approaches and usually analyze controlled or balanced data distribution.

**Table 6:** Comparative analysis of ARP spoofing detection methods.

References	Methods	Dataset	Accuracy (%)	Precision (%)	Recall (%)	*F*1-score (%)
Wang (2024)	CNN-BiLSTM hybrid model	ETI dataset (industrial IoT)	94.58	N/R	N/R	94.52
Vajrobol et al. (2025)	ML-based detection (RF, DT, XGBoost, and Hybrid)	CICIoT2023	95.10	95.20	95.10	95.10
Mvah et al. (2024a)	GaTeBaSep (game-theoretic ARP defense)	Mininet SDN simulation	97.00	N/R	N/R	N/R
El-Sayed et al. (2025)	SFARP multi-layer framework	CICIoMT2024, IoT datasets	98.30	97.60	98.90	98.20
Gafar et al. (2026)	DiMCA (P4 + ML SD-IoT framework)	CICIoMT2024, IoT datasets	99.22	N/R	N/R	99.76
(Ours)	SH-IORF framework (proposed)	CICIoMT2024 (ARP Spoofing)	99.90	99.95	99.83	99.89

Note: N/R indicates that the corresponding metric was not reported in the original publication. Direct numerical comparison is limited due to dataset heterogeneity; results for prior works are reported from their original publications and were evaluated on different datasets, whereas the proposed method is evaluated on the CICIoMT2024 ARP Spoofing subset with a strict three-way train/validation/test partitioning strategy with validation-based class weight estimation and threshold optimization.

The GaTeBaSep protocol (Mvah et al., 2024b) is a game-theoretic ARP spoofing defense method for SDN, which has been simulated with an accuracy of 97% in a game-based environment. Although this method enables adaptive attacker-defender modeling, it is tested only in simulated settings and cannot accurately reflect real IoMT traffic patterns. Recent SD-IoT models like SFARP (El-Sayed et al., 2025) and DiMCA (Gafar et al., 2026) show excellent detection performance with a 98.30% and 99.22% accuracy, respectively. These approaches combine programmable data-plane intelligence and distributed designs to augment detection and mitigation capabilities. Comparatively, under a rigorous three-way train/validation/test evaluation strategy with validation-based class weight estimation and threshold optimization, the proposed SH-IORF framework attains an accuracy of 99.90% and an *F*1-score of 0.9989 on the CICIoMT2024 ARP spoofing dataset. These outcomes suggest that the proposed framework shows high detection capability, and it has minimal limitations on false-negative behavior under IoMT intrusion detection conditions.

### 4.8 Cross-dataset generalization analysis

The proposed SH-IORF has been assessed utilizing the CICIDS2017 intrusion detection benchmark dataset provided by the Canadian Institute for Cybersecurity (Sharafaldin et al., 2018) to further test the strength of the framework beyond the CICIoMT2024 dataset. The purpose of this follow-up examination is to establish if the proposed SH-IORF framework is able to retain its classification capability in the presence of varying network traffic patterns and under different intrusion detection environments.

The CICIDS2017 is a popular intrusion detection dataset that consists of real-world enterprise-scale network traffic as well as a variety of attack categories that were collected under different operational conditions. For supplementary evaluation, the SH-IORF framework was utilized with the same architecture configuration and hyperparameters used in the main CICIoMT2024 experiments. To ensure methodological consistency between datasets, the validation-guided cost-sensitive learning strategy and threshold optimization procedure were retained.

Table 7 provides a comparative assessment of the CICIoMT2024 and CICIDS2017 based on the proposed SH-IORF framework. The overall accuracy, precision, recall, and *F*1-scores were 99.90%, 99.95%, 99.83%, and 99.89%, respectively, on the CICIoMT2024 dataset. For the CICIDS2017 dataset, the framework attains an accuracy of 99.32%, a precision of 99.28%, a recall of 99.14%, and an *F*1-score of 99.21%. Moreover, the framework showed excellent class separability on both datasets, AUC-ROC values of 0.9996 and 0.9986, respectively.

**Table 7:** Cross-dataset evaluation of the proposed SH-IORF framework.

Metric	CICIoMT2024	CICIDS2017
Accuracy	99.90%	99.32%
Precision	99.95%	99.28%
Recall	99.83%	99.14%
*F*1-score	99.89%	99.21%
AUC-ROC	0.9996	0.9986
False positive rate (FPR)	0.000417	0.0019
False negative rate (FNR)	0.0017	0.0086

The SH-IORF framework keeps the capability of intrusion detection stable across heterogeneous traffic and attack distributions, although moderate performance variation is found over the CICIDS2017 compared to CICIoMT2024. The outcomes confirm the validity of the system for several intrusion detection scenarios within a methodological framework of rigor and strict independence of tests.

## 5 Conclusion

This paper presented the SH-IORF framework for ARP spoofing detection in IoMT networks under realistic IoMT intrusion detection conditions. In order to overcome the limitations of previous evaluation designs, the following three-way stratified partitioning was applied, with the training, validation, and completely held-out testing dataset having the same class distribution across all subsets. Class penalty weights were estimated exclusively from the validation set, and the optimal classification threshold τ^*^ = 0.35 was determined by constrained optimization based only on validation probability outputs, without using the testing set when estimating the class weights, optimizing the classification threshold, or selecting the hyperparameters.

The proposed method was evaluated under a strict evaluation protocol on the CICIoMT2024 dataset, demonstrating an accuracy of 99.90%, a false negative rate of 0.17%, an *F*1-score of 0.9989, and an AUC-ROC of 0.9996. With a 2.9 × safety margin, the FNR of 0.17% meets the defined medical safety constraint of FNR ≤ 0.5%, giving only 40 missed detections of attacks on 23,390 attack samples and 12 false alarms across 28,768 benign samples. The results also show that the performance is consistent at different decision thresholds. The framework also proved to be computationally viable for deployment-oriented IoMT environments, with only around 32.8 s for complete training and a small serialized model size of 28.8 MB with low-latency inference behavior.

However, there are some drawbacks to this approach. The evaluation is mainly performed using the CICIoMT2024 dataset, but the diversity of IoMT network architectures and attack behaviors may not be fully representative in a heterogeneous clinical environment. In addition, the current framework only considers binary classification of ARP spoofing and does not explicitly extend to multi-attack intrusion detection or adversarial evasion strategies. The threshold optimization process is offline, so it may not be flexible enough in dynamic traffic environments. Furthermore, the framework is validated in an offline experimental environment and has not been validated on real IoMT devices or actual healthcare infrastructures; thus, further investigation is needed to validate interoperability and operational behavior in actual IoMT environments.

In addition, supplementary cross-dataset evaluation on the CICIDS2017 benchmark shows that the proposed SH-IORF framework also exhibits stable classification performance under different intrusion detection scenarios, which further demonstrates the reliability and generalization ability of the suggested model compared to the main experimental setting in the CICIoMT2024 benchmark.

The framework will be expanded to multi-class intrusion detection systems and generalized to IoMT data and scenarios in order to further investigate these ideas in future work. Traffic pattern responsive thresholds can be further enhanced by using an online or adaptive thresholding mechanism. Furthermore, there will be a need for system improvements along lines of adversarial robustness and lightweight deployment strategies for making the system more applicable in the safety-critical IoMT environment.

## Data Availability

The datasets analyzed in this study are publicly available from the Canadian Institute for Cybersecurity (CIC). The CICIoMT2024 dataset can be accessed at: https://www.unb.ca/cic/datasets/iomt-dataset-2024.html. Further inquiries can be directed to the corresponding author.
